# Influence of Chitosan Swelling Behaviour on Controlled Release of Tenofovir from Mucoadhesive Vaginal Systems for Prevention of Sexual Transmission of HIV

**DOI:** 10.3390/md15020050

**Published:** 2017-02-21

**Authors:** Fernando Notario-Pérez, Araceli Martín-Illana, Raúl Cazorla-Luna, Roberto Ruiz-Caro, Luis-Miguel Bedoya, Aitana Tamayo, Juan Rubio, María-Dolores Veiga

**Affiliations:** 1 Departamento Farmacia y Tecnología Farmacéutica, Facultad de Farmacia, Universidad Complutense de Madrid, 28040 Madrid, Spain; fnotar01@ucm.es (F.N.-P.); aracelimartin@ucm.es (A.M.-I.); racazorl@ucm.es (R.C.-L.); rruizcar@ucm.es (R.R.-C.); 2 Departamento Farmacología, Facultad de Farmacia, Universidad Complutense de Madrid, 28040 Madrid, Spain; lmbedoya@ucm.es; 3 Instituto de Cerámica y Vidrio, Consejo Superior de Investigaciones Científicas, 28049 Madrid, Spain; aitanath@icv.csic.es (A.T.); jrubio@icv.csic.es (J.R.)

**Keywords:** Human Immunodeficiency Virus, Acquired Immunodeficiency Syndrome, chitosan, mucoadhesive vaginal tablets, Tenofovir, controlled release, ex vivo bioadhesion, swelling behaviour, swelling witness microstructure

## Abstract

The main challenges facing efforts to prevent the transmission of human immunodeficiency virus (HIV) are the lack of access to sexual education services and sexual violence against young women and girls. Vaginal formulations for the prevention of sexually transmitted infections are currently gaining importance in drug development. Vaginal mucoadhesive tablets can be developed by including natural polymers that have good binding capacity with mucosal tissues, such as chitosan or guar gum, semisynthetic polymers such as hydroxypropylmethyl cellulose, or synthetic polymers such as Eudragit^®^ RS. This paper assesses the potential of chitosan for the development of sustained-release vaginal tablets of Tenofovir and compares it with different polymers. The parameters assessed were the permanence time of the bioadhesion—determined ex vivo using bovine vaginal mucosa as substrate—the drug release profiles from the formulation to the medium (simulated vaginal fluid), and swelling profiles in the same medium. Chitosan can be said to allow the manufacture of tablets that remain adhered to the vaginal mucosa and release the drug in a sustained way, with low toxicity and moderate swelling that ensures the comfort of the patient and may be useful for the prevention of sexual transmission of HIV.

## 1. Introduction

Acquired Immunodeficiency Syndrome (AIDS) continues to be one of the main public health problems around the world, especially in countries with the fewest resources. It is estimated that 36.7 million people are currently living with HIV [1]. The latest available data indicate that significant progress has been made over the last decade [2,3,4], and yet HIV continues to highlight the world’s inequalities. The main challenges in preventing HIV transmission are the lack of access to sexual education services, and sexual violence against young women and girls [5]. It is, therefore, necessary to have methods such as microbicides that are controlled by women themselves in order to prevent transmission, so they no longer depend on men to prevent the acquisition of the virus.

Tenofovir (TFV) is a drug that acts by blocking reverse transcriptase activity in HIV infection. It is currently being investigated for its potential microbicidal effect against HIV [6,7]. TFV microbicide formulations have had proven antiviral efficacy in animal models and are currently in phase III clinical trials. Recent studies have demonstrated that TFV vaginal administration has no significant cytotoxicity in women and that TFV has no toxicity for vaginal mucosa at concentrations commonly used as a microbicide [8,9]. Numerous reports have assessed and confirmed the effectiveness of TFV vaginal formulations. A wide range of dosage forms containing this drug have been evaluated, including gels [10,11], films [12,13], and intravaginal rings [14,15,16,17]. 

Solid formulations have the advantage of high dose accuracy and long-term stability, as compared to semi-solid systems. The polymers used in these formulations must, therefore, be able to adhere to the vaginal mucosa and modulate drug release from the dosage form. The term “adhesion” describes the ability of certain macromolecules to adhere to the body’s tissues; when this occurs in mucosa it is known as mucoadhesion. Although any material can adhere to the mucosa thanks to its viscous nature, there can be no real bioadhesion without an interrelation between some specific chemical groups in the polymers and biological tissues, or without establishing an interpenetration of chains. The dosage forms that bind to mucous membranes are described as mucoadhesive, as their purpose is to remain fixed at the point where the release and/or absorption of the drug occurs by prolonging its residence time [18,19].

All bioadhesive systems owe their properties to the inclusion of one or more types of polymeric molecules which, under appropriate conditions, establish interactions with the biological surface. One of these polymers is chitosan (CH), a natural, biocompatible, biodegradable, bioadhesive, and water-soluble polymer that degrades in acidic medium. It is obtained from the deacetylation of chitin, one of the most abundant polysaccharides in nature, as it is the structural element in the exoskeleton of crustaceans, such as crabs and shrimps. The amino and hydroxyl groups allow the adhesion to mucous through hydrogen bonds, and are protonated in an acid medium, which improves adhesion to negatively charged surfaces such as mucous. This polymer has been widely applied in the development of different pharmaceutical dosage forms for vaginal administration such as gels and tablets [20,21,22,23]. 

Possibly the most widely studied polymer for the development of such formulations is hydroxypropylmethyl cellulose (HPMC), a cellulose ether with methyl and hydroxypropyl groups used for the controlled release of drugs in hydrophilic matrix systems [24]. It is a FDA-approved polymer found in a wide range of applications, and was initially used in vaginal formulations as an excipient in the manufacture of films [25,26] and gels [27], although its use in vaginal administration tablets [20,28,29,30] has recently become more widespread. Another very similar polymer to HPMC and CH is guar gum (GG), which is also soluble in water, where it produces a viscous gel. GG is a biocompatible and biodegradable polysaccharide obtained from the seeds of *Cyamopsis tetragonoloba* used in the pharmaceutical industry as a binder or a disintegrant in tablets, and there are also several references to its use in the development of vaginal dosage forms. GG is sometimes present in bioadhesive vaginal gels, and has also been combined with HPMC to develop a bioadhesive vaginal tablet formulation [31,32]. All of the above-mentioned polymers are hydrophilic, and since the purpose of these formulations is their dissolution in the vaginal environment, it is also worth mentioning hydrophobic polymers such as Eudragit RS PO^®^ (ERS). This is a copolymer of ethyl acrylate, methyl methacrylate, and a low content of methacrylic acid ester with quaternary ammonium groups. The ammonium groups are present as salts and render the polymers permeable. It is insoluble in aqueous medium and has low permeability and pH-independent swelling [33]. Its inclusion in pharmaceutical forms of vaginal administration to date is much scarcer than for the polymers described above, and it is mainly used in nanocapsules, microspheres, and microparticles [21,34,35].

With this background, the aim of this study is to assess the potential of chitosan to develop sustained release mucoadhesive tablets of TFV, where the drug release from these systems depends on the properties of each polymer. These properties are also analysed in other natural, semisynthetic, and synthetic polymers in order to assess the advantages offered by CH in the development of these formulations.

## 2. Results and Discussion

### 2.1. Swelling Tests

**Figure 1:**
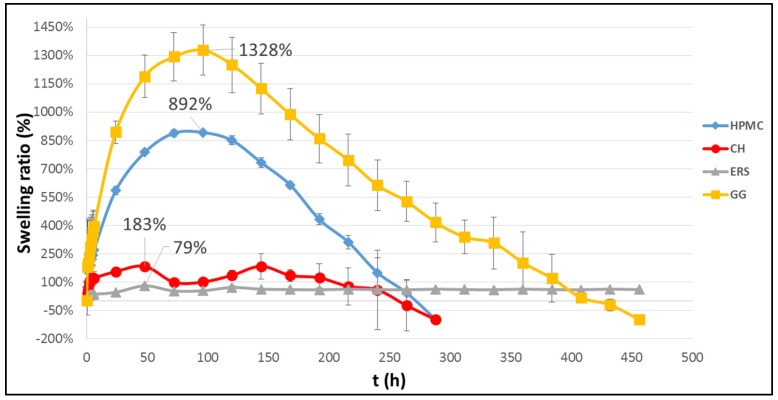
Swelling profile of each batch in simulated vaginal fluid (SVF). Data on the maximum swelling ratio (*SR*_max_) are indicated.

Figure 1 shows the swelling ratio (*SR*) profiles of the different batches studied. The maximum swelling ratio (*SR*_max_) is included for each swelling curve. The curves in Figure 1 show that swelling and erosion processes are present in most cases. Hydrophilic polymers such as CH, HPMC, and GG swell when in contact with an aqueous medium as opposed to disintegrating. These batches increase in size due to the relaxation of the polymer chains. A temperature of 37 °C causes a decrease in the vitreous transition temperature, forming an area where the polymers change from a crystalline to a rubbery state (known as the gel layer) [36]. It is, thus, possible to distinguish a first stage for the GG and HPMC batches in which the swelling process takes precedence until the *SR*_max_ (96 h) is reached, followed by the erosion of the formulations. HPMC and GG are significant for having a high SR; this is higher in the case of GG, which also takes much longer to dissolve completely. GG is well known for its high water-absorbent capacity, which is the reason it is used in the development of superabsorbent hydrogels [37].

**Figure 2:**
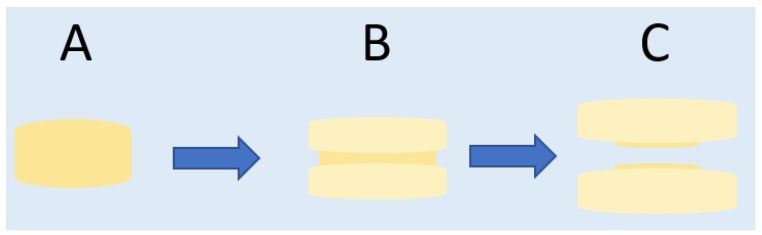
Chitosan compact swelling pattern. First the compact has a given shape (**A**), although the upper and lower layers swell in the presence of SFV, exerting pressure on the core (**B**) until finally this pressure causes the compact to break (**C**).

In contrast, CH undergoes moderate but sustained swelling and acquires an aqueous volume of 183% of its weight, corroborating the results of Chen et al. [38]. This is because CH has an unusual swelling process, and after about 48 h the pressure from the gel causes the breakdown of its core (Figure 2). From the time when the fracture occurs, the core portion which the gel prevented from swelling is exposed to the aqueous medium and absorbs water, causing a new increase in SR values (Figure 1). Finally, ERS is not a water-soluble polymer and hardly absorbs water from the medium—although the porous matrix adsorbs a small amount—and, thus, remains undissolved throughout the test (19 days). This renders it inadequate, as once the drug has been released, the compact would need to be removed. In view of the results, the batch with CH would be the most comfortable, since it undergoes moderate swelling and complete erosion. This factor, the comfort of women, is crucial for the adherence to the use of the formulation. In this respect there is no problem since studies show that vaginal tablets are the solid dosage form preferred by women for intravaginal administration [39]. In addition, the small size of the compacts developed (2.2–2.3 mm in height) makes them even more comfortable.

### 2.2. Release Study

**Figure 3:**
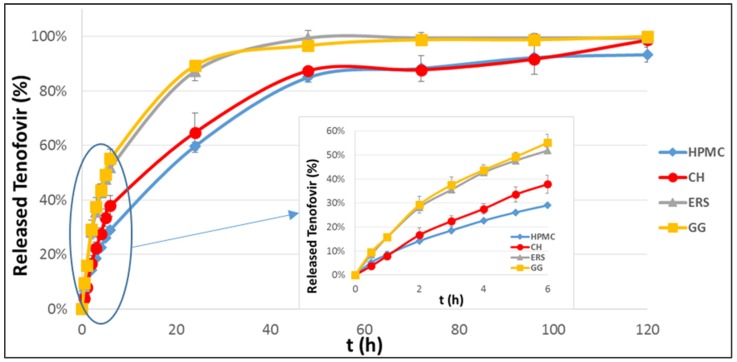
Tenofovir release profiles obtained from different batches in SVF.

A drug’s release rate from a dosage form can be influenced by different phenomena, ranging from drug dissolution to water absorption, polymer swelling, and the dissolution and diffusion of the drug through the polymer network [40]. Figure 3 shows the TFV release profiles corresponding to the prepared batches. The release data shows that HPMC and CH are the polymers that best control the drug release from the compact. One interesting result is that in the first 48 h these formulations released lower drug amounts than those containing ERS or GG, owing to the characteristics of ERS and GG. ERS is an insoluble polymer with pH-independent swelling and low permeability that is unable to gel in aqueous medium and, thus, barely controls the release of the drug [33]. Although GG produces the gel layer with the highest *SR* (Figure 1), it has very little consistency and the drug diffuses rapidly through it, so it does not represent a delayed release mechanism [41]. However, when HPMC and CH compacts are introduced into simulated vaginal fluid (SVF) the outer layers form a strong consistency gel, as reported by other authors, which controls TFV release [20]. This result would ensure women were protected against the transmission of HIV for at least 3–4 days (90%–95% TFV released). The inhibitory concentration 50 (IC_50_) of TFV has been found to be between 1.08–1.22 µM depending on the HIV strain used [42]. Thus, using these compacts IC_50_ is reached in a few minutes after administration.

In order to investigate the kinetics of TFV release from these formulations, mathematical model dependent methods (zero-order, first-order, Higuchi, Korsmeyer-Peppas, Hixson-Crowell, Hopfenberg and Weibull) were used to fit the experimental results. After analysing each batch, the models found to have the best fit to the curves in Figure 3 (high correlation coefficients *R*^2^) were Korsmeyer-Peppas and Higuchi and Weibull [43,44].

According to Korsmeyer-Peppas, the drug release as a function of time follows Equation (1): 

(1) $$M_{t}/M_{\infty }=Kt^{n}$$

Which can also be expressed as Equation (2):

(2) $$Ln\left(M_{t}/M_{\infty }\right)=Ln\left(K_{\mathrm{KP}}\right)+nLn\left(t\right)$$

where *M*_t_/*M*_∞_ is the fraction of drug released at time *t*, *K*_KP_ is a constant incorporating the structural and geometric characteristics of the compact, and *n* is the release exponent [44]. In the case of cylindrical compacts, depending on the *n* value the drug release could follow a pure diffusion process (*n* ≤ 0.45), an anomalous transport with simultaneous diffusion and structural modification of the polymer matrix (0.45 < *n* < 0.89), transport case II (*n* = 0.89) or transport Supercase II (*n* > 0.89). Both Case II and Supercase II involve the structural modification of the polymer matrix [43,45].

A good fit to the Higuchi model indicates that the drug diffuses through pores in the polymer matrix (this process is equivalent to Korsmeyer–Peppas for *n* ≤ 0.45). The Higuchi equation for fitting the curves of Figure 3 is in this case Equation (3), where *Q* is the amount of drug released in time *t* and *K*_H_ is the Higuchi dissolution constant:

(3) Q=KHt12

Finally, the curves in Figure 3 were also evaluated by the Weibull model, with the following mathematical equation:

(4) $$M=M_{0}\;\left[1-e^{-\;\frac{{\left(t-t_{lag}\right)}^{b}}{a}}\right]$$

where *M* is the dissolved drug, *M*_0_ is the total amount of drug in the compact and *t_lag_* is the lag time, *a* is a scale parameter that describes the dependence on time, and *b* describes the shape of the dissolution curve [43].

In our case, where there is no lag time (see curves in Figure 3), and because the drug release profiles have an exponential shape, *b* is equal to 1. If we take the constant *K*_W_ = 1/*a*, then Equation (4) can be summarised as Equation (5):

(5) $$\ln\left(1-\frac{M}{M_{0}}\right)=-K_{W}t$$

**Figure 4:**
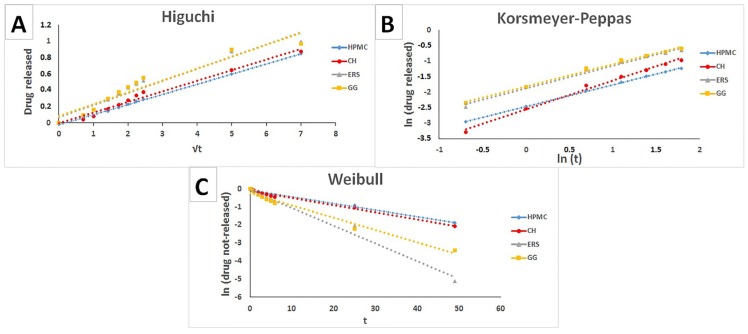
Fit of TFV release from batches HPMC, CH, ERS, and GG to the Higuchi (**A**) Korsmeyer-Peppas (**B**), and Weibull (**C**) models.

Figure 4 shows the corresponding fit of the experimental data to these drug release models, and Table 1 shows the *n*, *K*_KP_, *K*_H_, and *K*_W_ kinetic constants for these three models.

According to the data in Table 1, the batches with HPMC and CH have a good fit with the Higuchi kinetic (Figure 4A). However, the Higuchi kinetic can only be applied when the swelling and dissolution of the matrix are negligible [43], so it cannot be used to explain the drug release behaviour from these batches. All of the batches tested have a good fit to the Korsmeyer–Peppas kinetic (Figure 4B). The analysis of *n* values for different batches reveals that those prepared with HPMC, ERS and GG have similar values of close to 0.7, and that in the case of CH *n* is higher and close to 1. It can, therefore, be said that the TFV release from HPMC, ERS, and GG corresponds to an anomalous (non-Fickian) transport, while for CH—whose *n* value is over 0.89—it follows a Supercase II release, a mechanism that implies an extreme drug transport [45]. During polymer swelling the breakage of the compact occurs because the upper and lower layers of the compact swell to form a gel, causing a compressive stress on the core that prevents axial swelling. As the gel continues pressing on the core, the internal compact pressure increases until the core breaks (Figure 2) [46].

The *n* values for HPMC, ERS, and GG fall in the range 0.45–0.89, indicating that the drug release is governed by simultaneous structural modification and diffusion through the polymer matrix processes. In the HPMC and GG batches, the polymer swells at the same time as the drug diffuses through the gel formed. As has been shown in the swelling test, these two polymers form a long-lasting gel and the rearrangement of chains occurs slowly; the simultaneous diffusion is the process that causes the time-dependent anomalous effect [46].

ERS captures very little water, which rules out polymer swelling as a possible explanation. When the drug release from the ERS batch is fit to the Weibull model, the constant *K*_W_ is much higher than for the other polymers—including GG (Figure 4C)—although the differences between GG and ERS in the other models are insignificant (Figure 4A,B). The *K*w values in the Weibull equation represent the drug release rate constant. HPMC and chitosan have similar *K*_W_ values that are much lower than the other two formulations, signalling the greater control over the release of TFV from the compacts made with these polymers. In contrast, GG and ERS have much higher *K*_W_ values, since the drug also diffuses from these compacts at a greater rate. The Weibull model is used to analyse the release profile of matrix-type drug delivery, and this is the mechanism of TFV release in ERS. This is because ERS is a permeable polymer that allows water into the compact, followed by the dissolution of the drug in the medium, and finally the diffusion of TFV through the polymer.

### 2.3. Microstructure of Witnesses. FE-SEM, and Hg Porosimetry

**Figure 5:**
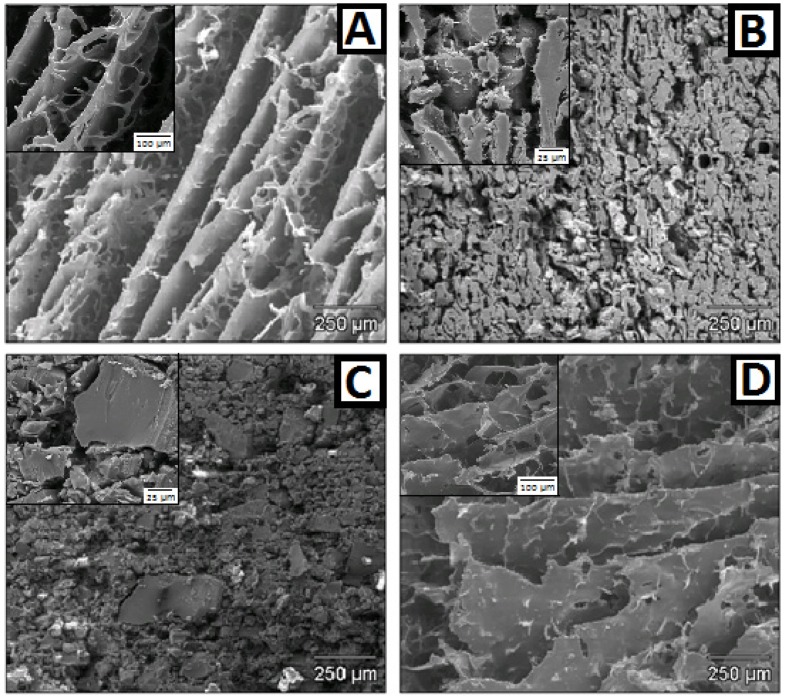
Electron microscopy micrographs of swollen witnesses of HPMC (**A**); chitosan (**B**); Eudragit^®^ RS PO (**C**); and guar gum (**D**).

It is well known that water is removed during freeze-drying of a hydrated polymer system, and the space that was originally occupied by the solvent is transformed into pores, generating a porous structure similar to a sponge [47]. As can be seen from the micrographs in Figure 5, the witness microstructures vary considerably depending on the polymer type. Figure 5A, corresponding to the swelling witness of HPMC, has a channelled microstructure formed when water enters the polymer during swelling. These channels allow the compacts to maintain their shape while the drug diffuses slowly between them. This perfectly homogeneous microstructure is maintained because HPMC swelling occurs progressively; the outer layers become swollen but the core remains unswollen until the outer gel erodes and water reaches the core [48]. This is observed in our release studies, thus, water mobility plays a role in controlling drug release. 

The micrograph of the CH witness (Figure 5B) shows a sponge-like microstructure with numerous pores in the polymer through which the SVF circulates, albeit with difficulty. This result explains the controlled release of TFV from CH in spite of its moderate swelling capacity. In contrast, no defined microstructure is observed for the ERS witness (Figure 5C); the formulation has a grainy microstructure with different-sized particles, but is unable to swell, which explains the failure of this formulation to control TFV delivery. Finally, the micrograph of the GG witness (Figure 5D) shows a perfectly microstructured formulation where the polymer is arranged in parallel sheets with the absorbed water between them. This microstructure explains why GG formulations swell the most and remain swollen the longest, as there is a high capacity for very effectively retaining water between these sheets. However, although the water cannot escape, the drug is able to diffuse through the polymer sheets, so their ability to retain the drug is minimal.

**Figure 6:**
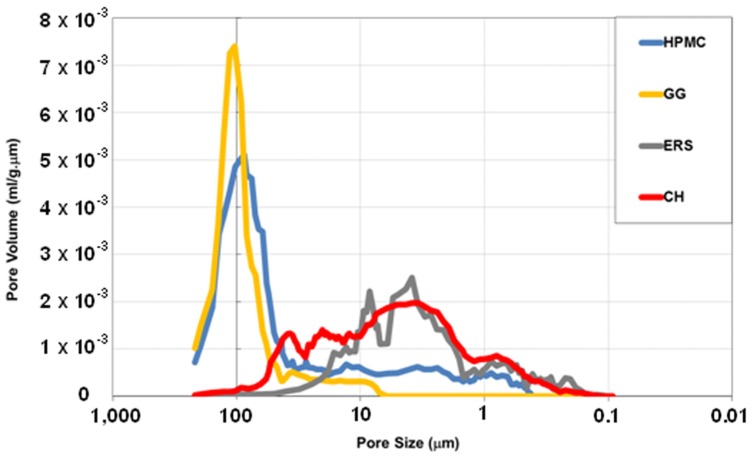
Pore distributions of HPMC, CH, ERS, and GG witnesses.

The above porous microstructures have been characterized by Hg porosimetry. Figure 6 shows the corresponding pore size distributions (PSD).

According to Figure 6, two types of PSD can be described: one has a narrow PSD where most of the pores are close to 100 μm, while the other has a wide PSD with pores of between 100 and 0.1 μm. Both PSD types are unambiguously associated to the swelling behaviours in Figure 1 and the FSEM micrographs in Figure 5. The HPMC and GG batches have high swelling characteristics with well-defined channelled microstructures and produce a narrow PSD with high pore sizes. In contrast, ERS and CH, with minimal swelling properties and grainy microstructures, produce a wide PSD with small pore sizes. There are two interesting observations: the first is that while the PSD of the GG batch also has a small number of pores with sizes between 50 and 10 mm, the PSD of HPMC also has small pores with sizes between 50 and 0.5 μm; and the other is that the PSD of CH has pores between 10 and 100 μm, while the PSD of ERS is below 50 μm. This points to the conclusion that as the compact has higher swelling properties, the corresponding PSD must have a high pore size.

Table 2 contains a summary of the results obtained from these PSD, and shows that mean pore size (*D*p) values are related to SR values, as mentioned earlier. Hence, the higher the swelling capacity, the higher the *D*p values. However, pore volumes (*V*p) are more closely related to the total number of pores present in the witness, so the lowest *V*p correspond to the ERS batch containing the smallest pore size. The CH witness has a higher *V*p value than ERS due to its larger pore size, as indicated by the *D*p. It is followed by GG, with a high *V*p value but lower than HPMC, although it has a higher *D*p. Finally, the highest *V*p corresponds to HPMC. HPMC’s higher *V*p compared to GG is due to the small pores of between 50 and 0.5 μm in HPMC, but not in GG. In contrast, pore area (*S*p) values show the opposite pattern; namely the higher the *D*p, the lower the *S*p. This is because pore area increases as pore volume decreases. As may be expected, porosity (P) values are related to *D*p and *V*p values, as porosity increases with both pore size and pore volume. Finally, bulk density (ρ_B_) values are related to *V*p and *P* values, as ρ_B_ corresponds to a sample where pores and material are measured as a whole. However, apparent density (ρ_A_) corresponds to the sample with no pores over 0.1 μm, i.e., a dense sample, and these values are characteristic of the chemical sample composition.

These results show that *P*, *V*p, *D*p, and ρ_B_ are related to the *SR*_max_ of the corresponding batches (Figure 1), but are not clearly related to the release profiles (Figure 3) or release kinetics (Table 1). Thus, the following relationship (with *R*^2^ = 0.992) has been found between *D*p and *SR*_max_:

(6) $$Dp=35.4\cdot Ln\left(SR_{\max}\right)+13.4\;$$

This equation indicates that for a polymer with a very low swelling capacity the release of the TFV drug in aqueous medium causes pores of around 13 μm. In our case the ERS polymer presented pores with a mean size of 9 μm, close to the value obtained by this equation.

### 2.4. Evaluation of Mucoadhesion

**Figure 7:**
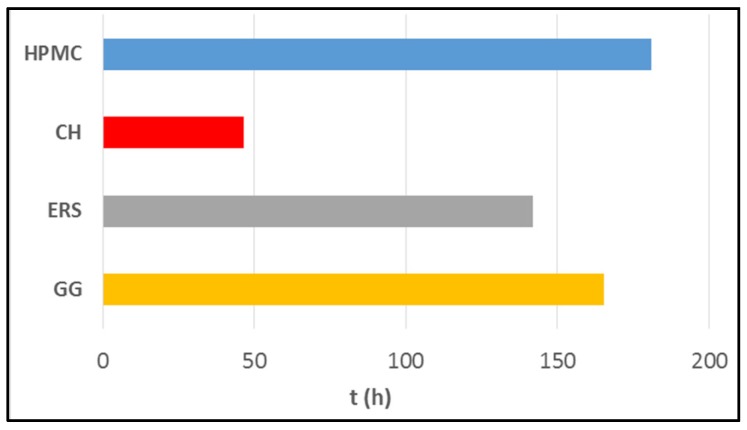
Mucoadhesion residence time of each batch in SVF.

An analysis of the mucoadhesion results (Figure 7) reveals that HPMC, ERS, and GG formulations remain attached to the mucosa for extended periods of time, even after all the TFV has been released. In contrast, the CH formulation shows a good initial adhesion to vaginal mucosa, and a residence time of about 48 h. This agrees with the results of other studies, highlighting the lower mucoadhesive ability of CH compared to cellulose derivatives [49]. This seems to be because the bonding to the mucosa by positively charged groups, as in the case of chitosan, is less durable than bonding through hydrogen bonds, which is typical of HPMC and GG.

The other three polymers show longer adhesion times to the mucosa of over 140 h in all cases. The formulation that remains attached for longest is HPMC, followed by GG and ERS. HPMC has been studied in depth, and this research corroborates its high mucoadhesive potential, caused by hydrogen bonding effects [50]. Although the mucoadhesive properties of GG have been poorly studied, another work shows its mucoadhesive strength is similar to HPMC [51]. Lastly, the most surprising results derive from the evaluation of the mucoadhesion of ERS, which in the previous literature is not classified as a mucoadhesive polymer. Our study shows that it has substantial mucoadhesive properties, and confirms a previous study comparing ERS with materials typically regarded in the literature as being good adhesives [52]. The adhesion to mucosa may be due to the presence of the quaternary ammonium group, which is protonated and may bind to negative charges in mucosa. Although these good mucoadhesion results highlight the high binding ability of the polymers, shorter times are required in this case—similar to the time for CH—since the TFV release occurs over a shorter period and there is no therapeutic justification for retaining the formulation adhered to the patient’s vaginal mucosa after the drug has been completely released. In addition to discomfort, it could induce the rejection of the formulation.

### 2.5. Cell Toxicity

The biocompatibility of the formulations was evaluated through an in vitro cellular toxicity assay. All of the components of the different formulations were incubated at 37 °C in a 5% CO_2_ atmosphere for five days before the assay to ensure that any potential toxic component would be present in the suspension. MT-2, a lymphoblastoid cell line, and HEC-1A, a uterus-derived cell line, were seeded and treated with different dilutions of the suspensions. All of the components were tested at a maximum concentration of 1000 μg/mL in base-5 serial dilutions. Experiments were performed on MT-2 cells to evaluate toxicity on the immune cells present in vaginal or uterine mucosae, and also on the uterus epithelial cell line (HEC-1A) to assess any potential damage to the integrity of the mucosae. Cytotoxic concentration 50 (CC_50_) were calculated when possible.

**Figure 8:**
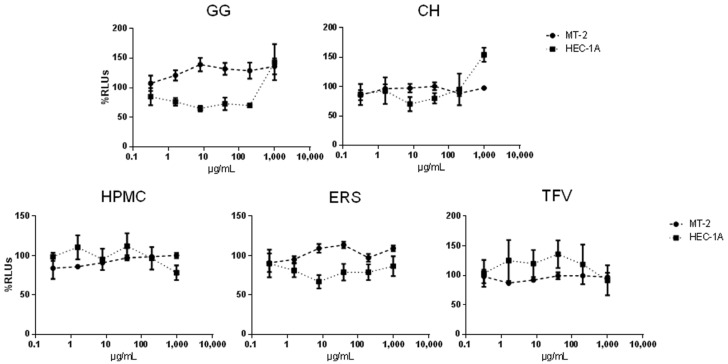
Cytotoxic evaluation of TFV, GG, CH, ERS, and HPMC measured in MT-2 cells and HEC-1A cells.

As shown in Table 3 and Figure 8, no toxicity was detected at the concentrations tested for any of the compounds. Interestingly, Tenofovir did not show cytotoxicity even at the highest tested concentration of 1000 µg/mL (around 3.3 mM).

## 3. Experimental Section

### 3.1. Materials and Preparation of the Compact

Tenofovir (TFV, lot: FT104801401, MW: 287.21 g/mol) was supplied by Carbosynth Limited (Berkshire, UK). Chitosan, with 97% deacetylation and a viscosity of 92 mpa·s (CH, lot: 8826900003), was provided by Nessler (Madrid, Spain). The molecular weight, 10^5^ g/mol, was estimated by viscometric measurements. Hydroxypropylmethylcellulose—Methocel^®^ K 100 M (HPMC; lot: DT352711, MW: 72 × 10^4^ g/mol) was kindly supplied by Colorcon Ltd. (Kent, UK). Eudragit RS^®^ (ERS; lot: G120238035, MW: 407.932 g/mol) was supplied by Evonik (Essen, Germany). Guar gum (GG; lot: SLBH5231V, MW: 22 × 10^4^ g/mol) was acquired from Sigma-Aldrich (Saint Louis, MO, USA). Magnesium stearate PRS-CODEX (MgSt; lot: 85269 ALP) was acquired from Panreac (Barcelona, Spain). All other reagents used in this study were of analytical grade and used without further purification. Demineralized water was used in all cases.

Four batches of compacts were prepared from physical mixtures of the corresponding polymer (HPMC, CH, ERS, GG), TFV and MgSt. In all cases, each compact contained 290, 30, and 3 mg of polymer, TFV, and MgSt respectively.

In all cases the compacts were prepared with a press similar to the one used for preparing solid samples for analysis by IR spectroscopy. A stainless-steel disc was placed in the die, and the physical mixture of the components was placed on top. A second stainless steel disc was then placed on top. Five tons of constant pressure was applied using a punch for four minutes. Finally, the piston and the discs were removed, and the compact was stored in a desiccator until its subsequent evaluation. The manufactured compacts were cylindrical in shape and measured 13 mm in diameter and 2.2–2.3 mm in height.

### 3.2. Methods

#### 3.2.1. Swelling Tests

The swelling pattern of the different batches in SVF were analysed using the method described by Ruiz-Caro et al. [53]. Each analysis was tested in triplicate. Swelling tests were carried out in a shaking water bath at 37 °C and 15 opm. In order to maintain the contact between the compact and the medium, and for more convenient handling of the samples during the test, each compact was previously fixed to a stainless steel disc, 3 cm in diameter, with a cyanoacrylate adhesive. At given time intervals (every hour during the first six hours and once a day for the remainder of the analysis), the discs were removed from the medium, placed on filter paper to eliminate the liquid excess and weighed. SR was calculated according to Equation (7):

(7) $$SR=\left(\frac{C_{s}-C_{d}}{C_{d}}\right)\cdot 100$$

where *C*_s_ and *C*_d_ correspond to the swollen and dry compact weights, respectively.

#### 3.2.2. Release Study

The method described by Sánchez-Sánchez et al. [20] was used concurrently with the swelling test to assess the release behaviour of TFV in each batch. Each sample was inserted in a borosilicate glass bottle containing 80 mL of the SVF [54] and placed in a shaking water bath (Selecta^®^ UNITRONIC320 OR, Barcelona, Spain) at 37 °C and 15 opm. Every day at given times, 5 mL samples were removed and filtered. The medium was replaced with the same volume of SVF at the same temperature. TFV release concentrations were quantified by UV spectroscopy at a wavelength of 260 nm in a Shimadzu^®^ UV-1700 spectrophotometer (Kyoto, Japan). The test was performed in triplicate in each case. Studies have been conducted on the solubility of TFV in SFV at room temperature and the results are 4 mg/mL. Therefore, this ensures that the release study is performed under sink conditions and the release of drug is not conditioned by its solubility but by the release system.

The drug release experimental data were fitted to different model-dependent methods (zero order, first order, Higuchi, Korsmeyer-Peppas, Hixson-Crowell, Hopfenberg, and Weibull models) to investigate the kinetics of drug release from the various batches [43].

#### 3.2.3. Swelling Witnesses

In order to characterize the microstructure acquired by the compacts when introduced in the SVF, swelling witnesses were prepared by determining the time each batch reached the maximum SR. This was done by attaching two compacts from each batch to the same stainless steel discs used for the swelling test. The discs were treated in the same way as in the swelling test. They were immersed in a beaker with SVF which was then placed in the shaking water bath (Selecta® UNITRONIC320 OR, Barcelona, Spain) (37 °C and 15 opm). The compacts were left under these conditions until they reached the maximum SR. Each compact was then extracted from the medium and lyophilized, and then stored in a desiccator until analysis. Witness microstructures were analysed by electron microscopy using a field emission scanning electron microscope (FE-SEM, Hitachi 4700, Tokyo, Japan) at an accelerating voltage of 15 V. Pore size distributions (PSD) were determined by mercury porosimetry using an Autopore II 9215 (Micromeritics Corp., Norcross, GA, USA). The corresponding pore volumes (*V*p), pore areas (*S*p), mean pore sizes (*D*p), bulk and apparent densities (ρ_B_, ρ_A_), and porosities (*P*) of the samples were calculated from these PSD, assuming cylindrical pore shapes in all cases.

#### 3.2.4. Assessment of Mucoadhesion

A new ex vivo mucoadhesion test was used to determine how long the compact remained adhered to the vaginal mucosa. A sample of freshly excised veal vaginal mucosa (obtained from a local slaughterhouse) was fixed with a cyanoacrylate adhesive to an 8.5 cm × 5 cm stainless steel plate (SSP). Each compact was then adhered to the mucosa, applying a given pressure (500 g for 30 s). The SSP was placed at an angle of 60° inside a beaker containing 150 mL of SVF, and this system was inserted in the shaking water bath (Selecta^®^ UNITRONIC320 OR, Barcelona, Spain) at 37 °C and 15 opm. The bioadhesion time of each batch was assessed by visual observation of the samples. All batches were tested in duplicate.

#### 3.2.5. Cytotoxicity Assessment

Two human cell lines were used: a lymphoblastic cell line, MT-2 [55] and a uterus/endometrium epithelial cell line, HEC-1-A (kindly provided by M. A. Muñoz, Hospital Gregorio Marañón, Madrid, Spain). Both cells were grown in RPMI 1640 medium supplemented with 10% (*v*/*v*) foetal bovine serum, 2 mM l-glutamine and 50 μg/mL streptomycin at 37 °C with a humidified atmosphere of 5% CO_2_. HEC-1-A cells were detached by treatment with a trypsin 0.25% and EDTA 0.03% solution. Cell cultures were split twice a week.

Cell toxicity was measured by the CellTiter Glo viability assay (Promega). Briefly, GG, CH, ERS, HPMC, and TFV were suspended in water at a concentration of 10 mg/mL and left in culture (5% CO_2_ and 37 °C) for five days [56]. Cells were then seeded in 96 microwell plates at a density of 1 × 10^5^ cells per well in the case of MT-2, and 2 × 10^4^ in the case of HEC-1-A, in complete RPMI medium, and treated with fresh medium containing different concentrations of suspensions (1000, 200, 40, 8, 1.6, and 0.32 µg/mL), or with the same concentration of vehicle (water). After 48 h of incubation, cell viability was measured following the manufacturer’s instructions (CellTiter Glo viability assay), and RLUs were obtained in a luminometer. Data were normalized using RLUs obtained from cells treated with vehicle (100%) as a reference. Values of CC_50_ were calculated using GraphPad Prism Software (non-linear regression, log inhibitor versus response).

## 4. Conclusions

Vaginal compacts can be a very useful tool for the prevention of HIV transmission from men to women. Adherence to the use of microbicides has been one of the main drawbacks in demonstrating the efficacy of the formulations studied to date. In contrast, these compacts would decrease the frequency of administration by achieving sustained release of the drug over several days, resulting in a greater adherence to the treatment.

From the results obtained it can be concluded that there are two polymers (CH and HPMC) with the potential to achieve sustained and complete release of TFV from vaginal mucoadhesive compacts. However, the good bioadhesive properties of CH, which allow the formulation to remain attached to the vaginal mucosa only until all of the drug has been released, its moderate SR, which ensures more comfort for the patient than the other polymers tested, and its low cytotoxicity warrants that CH compacts containing TFV have proven to be a suitable formulation for the prevention of sexual transmission of HIV.

## Figures and Tables

**Table 1:** TFV release kinetics from HPMC, CH, ERS, and GG batches.

Batch	Korsmeyer-Peppas			Higuchi		Weibull	
	*K*_KP_	*n*	*R*^2^	*K*_H_	*R*^2^	*K*_W_	*R*^2^
HPMC	0.088	0.63	0.9899	0.124	0.9980	0.036	0.9931
CH	0.077	0.92	0.9926	0.130	0.9815	0.040	0.9839
ERS	0.152	0.73	0.9887	0.148	0.9453	0.098	0.9859
GG	0.161	0.71	0.9929	0.145	0.9231	0.068	0.9756

**Table 2:** Pore volume (*V*p), pore area (*S*p), mean pore size (*D*p), bulk and apparent densities (ρ_B_, ρ_A_), and porosity (*P*) of HPMC, CH, ERS, and GG witnesses.

Witness	*V*p (cm^3^·g^−1^)	*S*p (m^2^·g^−1^)	*D*p (μm)	ρ_B_ (cm^3^·g^−1^)	ρ_A_ (cm^3^·g^−1^)	*P* (%)
HPMC	5.97	0.36	91.89	0.14	0.90	84
CH	1.74	0.43	28.74	0.38	1.19	67
ERS	0.35	3.59	9.16	0.77	1.06	27
GG	5.89	0.25	106.08	0.14	0.97	85

**Table 3:** CC_50_ values of TFV, GG, CH, ERS, and HPMC obtained from the cytotoxicity assay in both MT-2 and HEC-1A cell lines. CC_50_: cytotoxic concentration 50%.

Evaluated Substance	Cell Line	CC50
**TFV**	MT-2	>1000 µg/mL
	HEC-1A	>1000 µg/mL
**GG**	MT-2	>1000 µg/mL
	HEC-1A	>1000 µg/mL
**CH**	MT-2	>1000 µg/mL
	HEC-1A	>1000 µg/mL
**ERS**	MT-2	>1000 µg/mL
	HEC-1A	>1000 µg/mL
**HPMC**	MT-2	>1000 µg/mL
	HEC-1A	>1000 µg/mL
